# The Effects of* Aloe vera* Cream on the Expression of CD4^+^ and CD8^+^ Lymphocytes in Skin Wound Healing

**DOI:** 10.1155/2018/6218303

**Published:** 2018-02-15

**Authors:** Yos Adi Prakoso, Kurniasih

**Affiliations:** ^1^Faculty of Health, Muhammadiyah University of Sidoarjo, Sidoarjo, East Java, Indonesia; ^2^Department of Pathology, Faculty of Veterinary Medicine, University of Gadjah Mada, Yogyakarta, Indonesia

## Abstract

The aim of this study is to explore the effect of topical application of* Aloe vera* on skin wound healing. Thirty-six male Sprague-Dawley rats weighing 150–200 grams were divided into four groups. All groups were anesthetized, shaved, and exposed to round full-thickness punch biopsy on the back: group I (control); group II (treated with 1%* Aloe vera* cream); group III (treated with 2%* Aloe vera* cream); and group IV (treated with madecassol®). The treatments were given once a day. Macroscopic and microscopic examination were observed at 5, 10, and 15 days after skin biopsy. Skin specimens were prepared for histopathological study using H&E stain and IHC stain against CD4^+^ and CD8^+^ lymphocytes. All the data were analyzed using SPSS16. The result showed that topical application of 1% and 2%* Aloe vera* cream significantly reduced the percentage of the wound, leucocytes infiltration, angiogenesis, and expression of CD8^+^ lymphocytes and increased the epidermal thickness and the expression of CD4^+^ lymphocytes (*p* ≤ 0,05). There was no significant difference in the number of fibroblasts in all groups. Topical application of 1% and 2%* Aloe vera* cream has wound healing potential via their ability to increase the ratio of CD4^+^/CD8^+^ lymphocytes in the wound area.

## 1. Introduction

*Aloe vera* is the one of potential herbal medicines and could be found in the tropical area [1], such as Indonesia.* Aloe vera* leaf contains gel [2]. Its gel contains flavonoid, terpenoid, lectin, anthraquinone, tannin, and saponin. And these contents have pharmacological properties to promote wound [3] and skin burns healing [4] and have antibacterial [5], anti-inflammatory [6], and cosmetic characteristics; this gel could also be used as a moisturizer and in dermatological products [7].

Damage to the normal anatomical structure and function of tissues is defined as wound [8]. Normally, each wound will be healed through a process involving hemostasis, inflammation, proliferation, maturation, and remodeling [8, 9]. T lymphocytes, especially CD4^+^, play important role in wound healing by secreting lymphokines to activate fibroblast, as wound anti-inflammatory agent, and activate factor in major histocompatibility complex (MHC) class II. On the other hand, CD8^+^ lymphocytes play a role as proinflammatory and activate factor in MHC class I [10]. Effect of* Aloe vera* on wound healing, especially role of CD4^+^ and CD8^+^ lymphocytes, is not fully known. In this study, the effects of* Aloe vera* on the percentage of the wound area, epidermal thickness, neutrophil infiltration, fibroblast, angiogenesis, and CD4^+^ and CD8^+^ lymphocytes were observed.

## 2. Material and Methods

This study was taken in Laboratory of Pathology, Faculty of Veterinary Medicine, Gadjah Mada University. All animal procedures in this study were approved by ethical clearance committee of Gadjah Mada University, with license numbers 00052/04/LPPT/VII/2016.* Aloe vera* from Kalimantan, Indonesia, was used in this study.* Aloe vera* leafs were peeled, cut, put in a blender mix, and extracted by alcohol 70%.* Aloe vera* extract was made into 1% and 2% cream concentration. The cream was made from the mixture of* Aloe vera* extract, stearic acid, potassium hydroxide (KOH), glycerin, methyl paraben, propylparaben, and water [11].

Thirty-six male Sprague-Dawley rats weighing 150–200 grams were divided into four groups. All groups were shaved and exposed to round 4 mm full-thickness punch biopsy on the back under ketamine 50 mg/kg BW and xylazine 4 mg/kg BW anesthesia. Next, group I was the control group; group II was treated with topical application of 1%* Aloe vera* cream; group III was treated with 2%* Aloe vera* cream; and group IV was treated with madecassol. The treatments were given once a day, for fifteen days. Three rats from each group were euthanized with cervical dislocation on days 5, 10, and 15 to collect skin specimen. The collected specimen was stored in neutral buffer formalin 10%, dehydrated, blocked in paraffin, and cut using microtome for H&E and IHC stain. IHC stain used monoclonal antibodies for CD4^+^ (anti-rat CD4^+^, Novocastra, RTU-CD4-1F6, Cat. number PA0427) and CD8^+^ (anti-rat CD8^+^, Novocastra, RTU-CD8-295, Cat. number PA0183).

The data was divided into macroscopical and microscopical data. Macroscopical data was the percentage of the wound area. Microscopical data was epidermal thickness, neutrophils infiltration, fibroblast, angiogenesis, and CD4^+^ and CD8^+^ lymphocytes. Microscopical examination was performed by ImageJ software. All data were analyzed by SPSS 16. Epidermal thickness, neutrophils infiltration, fibroblast, angiogenesis, and CD8^+^ lymphocytes data were analyzed with Kruskal-Wallis and Mann–Whitney *U* Test. CD4^+^ lymphocytes were analyzed with two-way ANOVA and Post hoc test.

## 3. Result and Discussion

**Figure 1:**
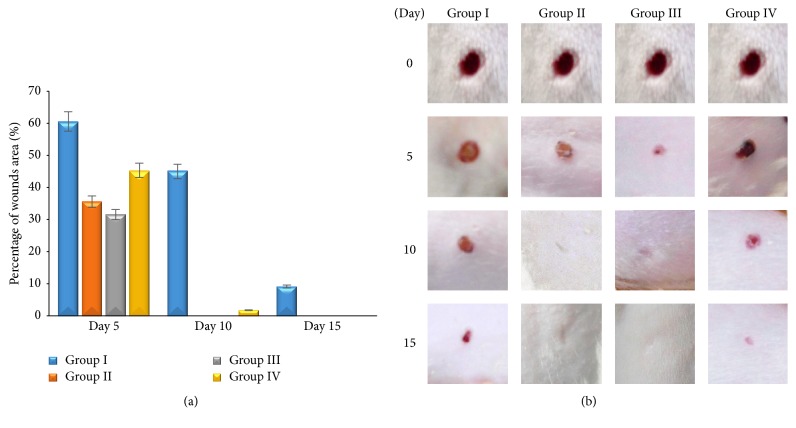
Percentage of wounds area (a); macroscopical examination of wound area from each group (b).

**Figure 2:**
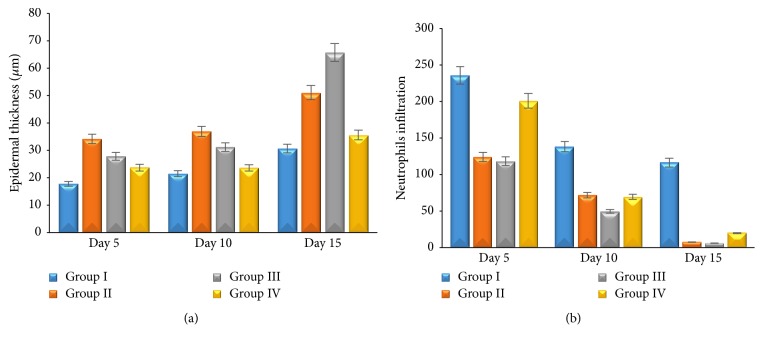
The average thickness of epidermis (a); the average number of neutrophils infiltration (b) on skin wound.

**Figure 3:**
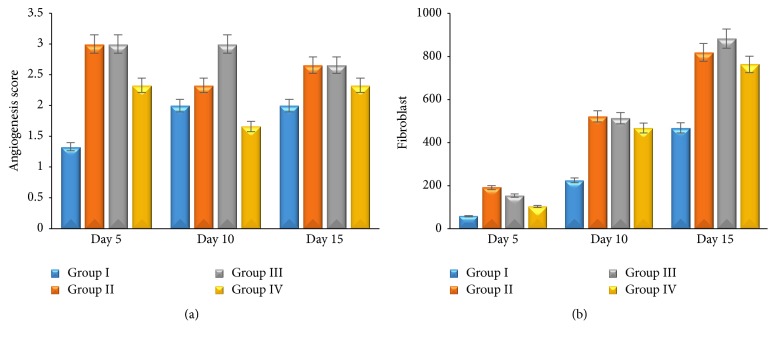
The average score of angiogenesis (a); the average number of fibroblast (b) on skin wound.

There is significant difference between group II, group III, and group IV compared with group I (*p* ≤ 0,05). It shows that topical application of 1% and 2%* Aloe vera* cream and madecassol has potential effect on wound healing to the percentage of wounds area (Figures 1(a) and 1(b)). All groups showed that epidermal thickness increased; however, neutrophils infiltration and angiogenesis decreased during the wound healing process. Group II and group III showed significant epidermal thickness increasing (*p* ≤ 0,05) (Figure 2(a)) and neutrophils infiltration decreasing (*p* ≤ 0,05) (Figure 2(b)), as well as angiogenesis compared with the other group (*p* ≤ 0,05) (Figure 3(a)). Proliferating of fibroblast did not show the significant difference in all groups (*p* ≥ 0,05). Fibroblast increased significantly during the wound healing process (*p* ≤ 0,05) (Figure 3(b)). This result shows that 1% and 2%* Aloe vera* cream has better effect than madecassol against the epidermal thickness, neutrophil infiltration, and angiogenesis; however, it was not different regarding fibroblast.

**Figure 4:**
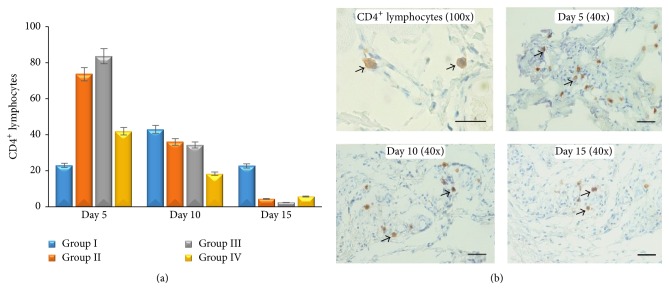
The average number of CD4^+^ lymphocytes (a); microscopical examination of CD4^+^ lymphocytes on skin wound (IHC antibody anti CD4^+^, DAB, scale bar 50 *μ*m).

**Figure 5:**
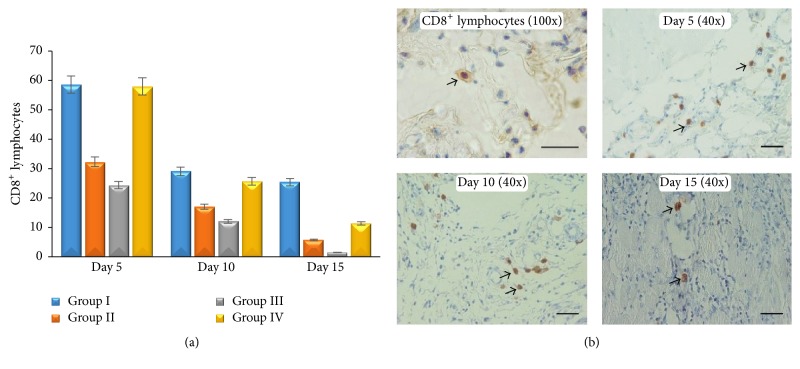
The average number of CD8^+^ lymphocytes (a); microscopical examination of CD8^+^ lymphocytes on skin wound (IHC antibody anti CD8^+^, DAB, scale bar 50 *μ*m).

CD4^+^ lymphocytes in this study showed that there is no difference between all groups (*p* ≥ 0,05), although CD4^+^ lymphocytes in group II and group III increased maximally on day 5 compared with other groups. CD4^+^ lymphocytes in group I increased on day 10, while the other groups decreased (Figure 4(a)). Low CD4^+^ lymphocytes infiltration in group I has an adverse effect on wound healing when compared with the others. In every examination period (days 5, 10, and 15), group I and group IV showed higher CD8^+^ lymphocytes infiltration compared with group II and group III (*p* ≤ 0,05). It proved that* Aloe vera* has a better effect on decreasing CD8^+^ lymphocytes infiltration in the wound area. Nevertheless, there is a sequential decreasing CD8^+^ lymphocytes infiltration in all groups (Figure 5(a)). Both CD4^+^ and CD8^+^ lymphocytes show a trend of decreasing when it approaches the end of the healing period (*p* ≤ 0,05) (Figures 4 and 5). This study showed that* Aloe vera* may shorten healing period by increasing CD4^+^/CD8^+^ lymphocytes infiltration ratio earlier on wound tissue.

*Aloe vera* contains anthraquinone, sterol, and saponin. Those active ingredients have a role as antibacterial and wound healing promotor [5]. Topical application of* Aloe vera* cream increases the number of CD4^+^ lymphocytes and decreases CD8^+^ lymphocytes. Increasing the number of CD4^+^/CD8^+^ lymphocytes ratio in group II and group III induces the other healing factors to promote wound [12].* Aloe vera* application on wound has a potential effect as the anti-inflammatory agent. Its ability decrease leucocytes adhesion on wound via cyclooxygenase and prostaglandin route [13]. The inflammatory brief period during wound will make faster healing process to the next phase. On proliferation phase, CD4^+^ lymphocytes induce keratinocytes to release IL-1 in the wound area. Keratinocytes have a potential role on epithelization, proliferation, and maturation of epidermis [14]. IL-1 that has been released by keratinocytes induces endothelial cells to form angiogenesis and fibroblast to form extracellular matrix [15].

Angiogenesis was formed to supply nutrition and others healing factors to wound area [16]. Failure of angiogenesis impaired wound healing [17]. In this study, increasing number of CD4^+^/CD8^+^ lymphocytes ratio in group II and group III activates angiogenesis maximally on day 5 and it makes healing faster. Angiogenesis mediates fibroblast migration on wound tissues. Fibroblast-like cells appeared in group II and group III on day 5. Fibroblast-like cells are one indicator that healing process occurred faster [18]. It proves that* Aloe vera* has a potential effect on healing. Increasing of fibroblast as a result of lymphokine induced which secreted by CD4^+^ lymphocytes [19]. CD4^+^ lymphocytes have a role as healing promotor to cellular immune response [20]. CD4^+^ lymphocytes depletion can decrease skin tension and extracellular matrix component [21]. On the other hand, CD8^+^ lymphocytes have a role as proinflammatory agent [22] and as pain receptor. Increasing of CD8^+^ lymphocytes impaired wound healing. In this study, decreasing of CD8^+^ lymphocytes on wound area was followed by increasing of CD4^+^ lymphocytes. Decreasing of CD8^+^ lymphocytes on wound area can decrease IL-3, so that healing process will be faster [23]. Healing process could be identified macroscopically by percentage of wound area (Figure 1(b)).

## 4. Conclusion

*Aloe vera* has a potential effect to increase the ratio of CD4^+^/CD8^+^ lymphocytes, which is mechanism influence on wound healing by decreasing percentage of the wound area, neutrophils infiltration, and angiogenesis and, however, increasing epidermal thickness and fibroblast in wound tissue. 1% and 2%* Aloe vera* cream have similar potential effect and both of 1% and 2%* Aloe vera* cream can be used as alternative therapy on the wound.
